# Author Correction: Cytokinin depends on GA biosynthesis and signaling to regulate different aspects of vegetative phase change in Arabidopsis

**DOI:** 10.1038/s41467-025-64338-6

**Published:** 2025-09-24

**Authors:** Sören Werner, Danuše Tarkowskà, Thomas Schmülling

**Affiliations:** 1 Institute of Biology/Applied Genetics, Dahlem Centre of Plant Sciences (DCPS), Freie Universität Berlin, Albrecht-Thaer-Weg 6, Berlin, Germany; 2 Laboratory of Growth Regulators, Institute of Experimental Botany, Czech Academy of Sciences and Faculty of Sciences, Palacký University, Šlechtitelů 27, Olomouc, Czech Republic

Correction to: *Nature Communications* 10.1038/s41467-025-61507-5, published online 08 July 2025

In the version of this article initially published, in the *y*-axis labels of the graphs in Fig. 4 and Supplementary Fig. 3 and in the legend of Supplementary Table 1, units were listed as “nmol” and should have read “fmol”. This has been corrected in the HTML and PDF versions of the article.

