# Supplementary material for: Cytokinin depends on GA biosynthesis and signaling to regulate different aspects of vegetative phase change in Arabidopsis
Source: Nat Commun. 2025 Jul 8;16:6292. doi: 10.1038/s41467-025-61507-5 (PMC12238502; doi:10.1038/s41467-025-61507-5)
Supplement: Supplementary file 1 — Supplementary information [file 41467_2025_61507_MOESM1_ESM.pdf]

SUPPLEMENTARY INFORMATION

Cytokinin depends on GA biosynthesis and signaling to regulate different aspects vegetative phase change in *Arabidopsis*

Sören Werner[1], Danuše Tarkowskà[2] & Thomas Schmülling[1,*]

[1]Institute of Biology/Applied Genetics, Dahlem Centre of Plant Sciences (DCPS), Freie Universität Berlin, Albrecht-Thaer-Weg 6, 14195 Berlin, Germany

[2]Laboratory of Growth Regulators, Institute of Experimental Botany, Czech Academy of Sciences and Faculty of Sciences, Palacký University, Šlechtitelů 27, 78371 Olomouc, Czech Republic

*Corresponding author, email: t.schmuelling@fu-berlin.de

List of supplementary material

Supplementary Figures 1-7
Supplementary Tables 1-4
Supplementary References

Supplementary Figures

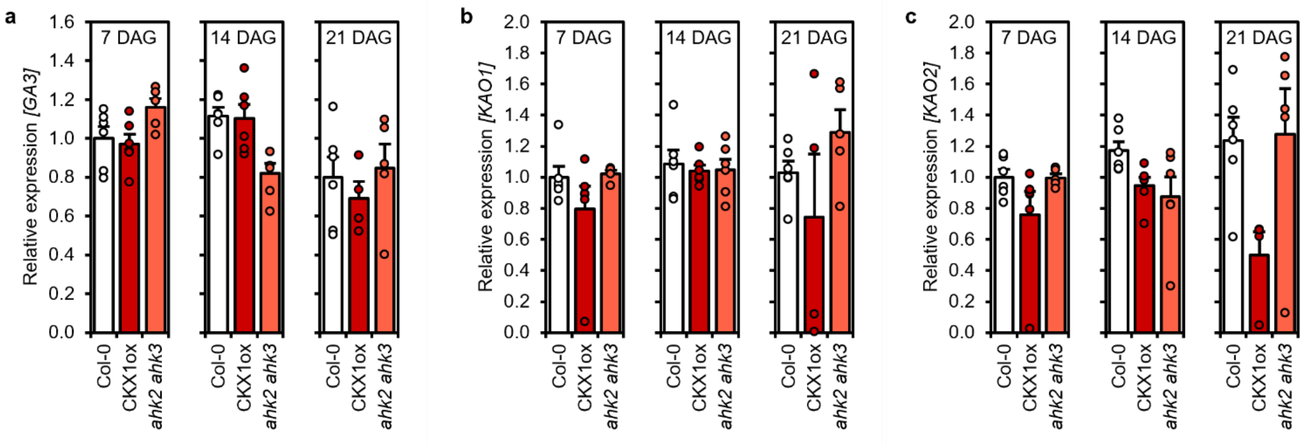

Supplementary Figure 1. GA biosynthesis genes with unchanged expression in CK-deficient plants. a-c Expression of GA biosynthesis genes in whole shoots of SD-grown Col-0, *ahk2,3* and CKX1ox plants. Numbers of biological replicates: Col-0 ($n_7 = 6$; $n_{14} = 6$; $n_{21} = 6$), CKX1ox ($n_7 = 6$; $n_{14} = 6$; $n_{21} = 4$), *ahk2 ahk3* ($n_7 = 5$; $n_{14} = 6$; $n_{21} = 5$). Transcript levels were determined by qRT-PCR. Data were normalized to *TAFII15* and *PP2AA2*. Data displayed are expressed as mean ± SEM. Dots indicate each single biological replicate. No statistically significant differences were observed compared to the wild type of the respective time point, as calculated by one-way ANOVA, post-hoc Dunnett's test.

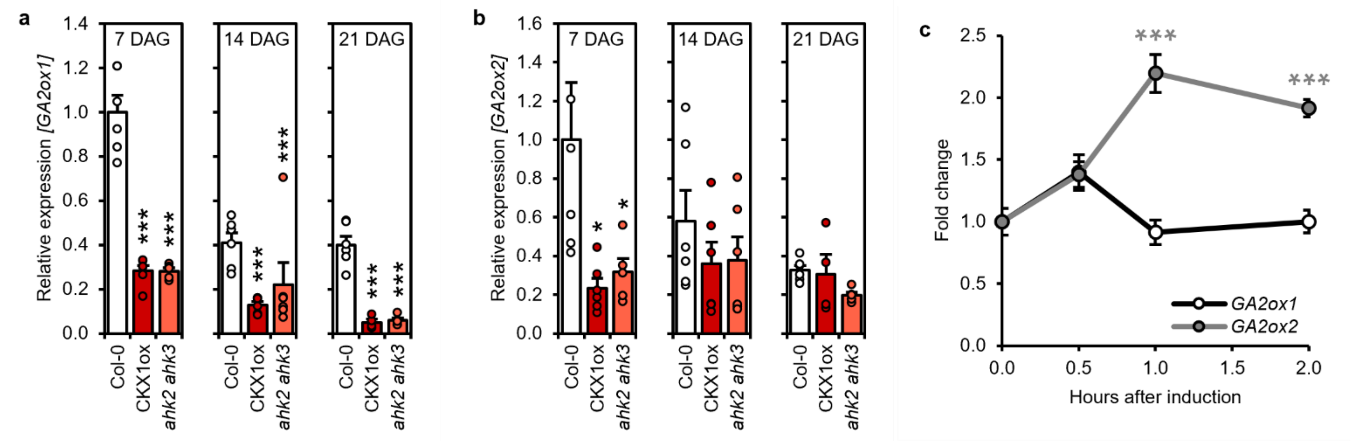

Supplementary Figure 2. Expression of GA catabolism genes is altered by CK. a-b Expression of GA catabolism genes in whole shoots of SD-grown Col-0, *ahk2,3* and CKX1ox plants. Numbers of biological replicates: Col-0 ($n_7 = 6$; $n_{14} = 6$; $n_{21} = 6$), CKX1ox ($n_7 = 6$; $n_{14} = 6$; $n_{21} = 4$), *ahk2 ahk3* ($n_7 = 5$; $n_{14} = 6$; $n_{21} = 5$). **c** Expression kinetics of GA catabolism genes in 10-day-old SD-grown wild-type seedlings after treatment with 1 µM BA (n = 6 biological replicates). Transcript levels were determined by qRT-PCR. Data were normalized to *TAFII15* and *PP2AA2*. Data displayed are expressed as mean ± SEM. Dots indicate each single biological replicate. Asterisks indicate statistically significant differences compared to the wild type of the respective time point (**a, b**), or compared to time point 0 (**c**), as calculated by one-way ANOVA, post-hoc Dunnett's test (*$p < 0.05$; **$p < 0.01$; ***$p < 0.001$).

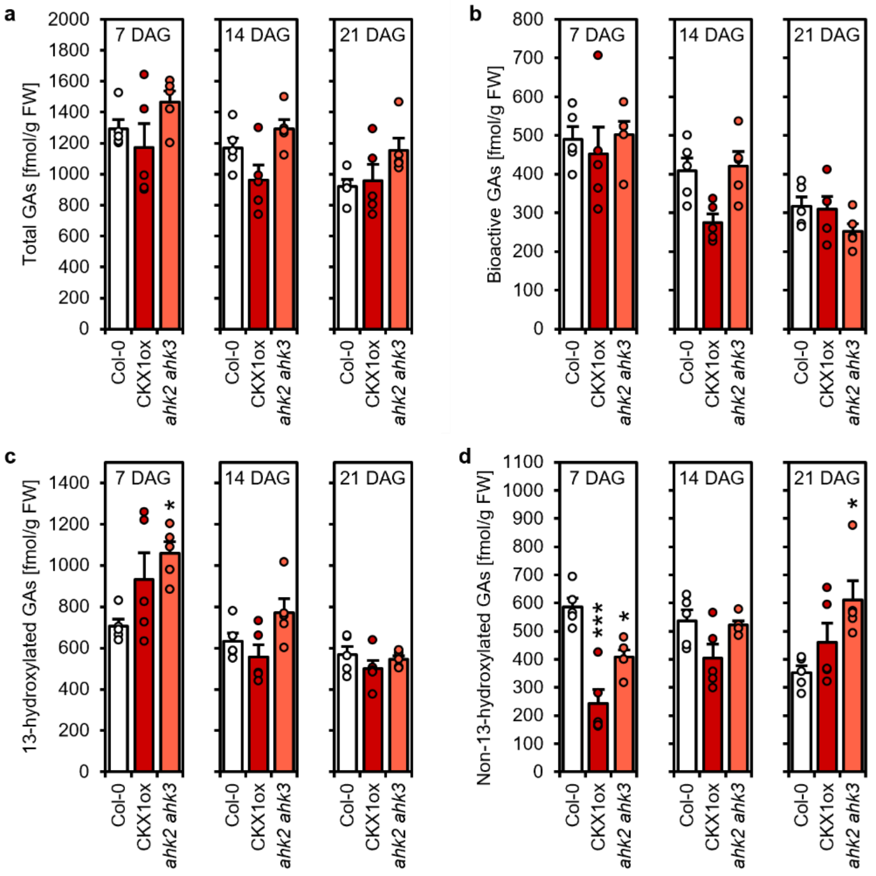

Supplementary Figure 3. Concentration of GA metabolites in CK-deficient plants. Shown are concentrations of total GAs (**a**), bioactive forms of GA (**b**), 13-hydroxylated GAs (**c**) and non-13-hydroxylated GAs (**d**) in shoots of SD-grown plants (n = 5 biological replicates). Data displayed are expressed as mean ± SEM. Dots indicate each single biological replicate. Asterisks indicate statistically significant differences compared to the wild type of the respective time point, as calculated by Kruskal-Wallis test (*$q < 0.05$; **$q < 0.01$; ***$q < 0.001$).

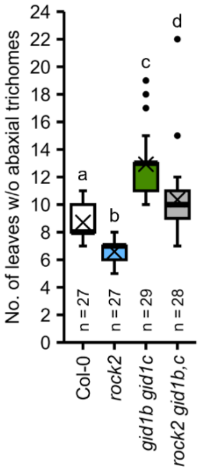

Supplementary Figure 4. Number of juvenile leaves in hybrid plants of *rock2* and the GA receptor mutant *gid1b gid1c*. Shown are the number of leaves without abaxial trichomes of SD-grown *rock2 gid1b,c* hybrid plants in comparison to their parents and wild type. In box plots, the center line represents the median value and the boundaries indicate the 25th percentile (upper) and the 75th percentile (lower). The X marks the mean value. Whiskers extend to the largest and smallest value, excluding outliers which are shown as dots. Letters indicate statistically significant differences between the genotypes, as calculated by Kruskal-Wallis test ($q < 0.05$).

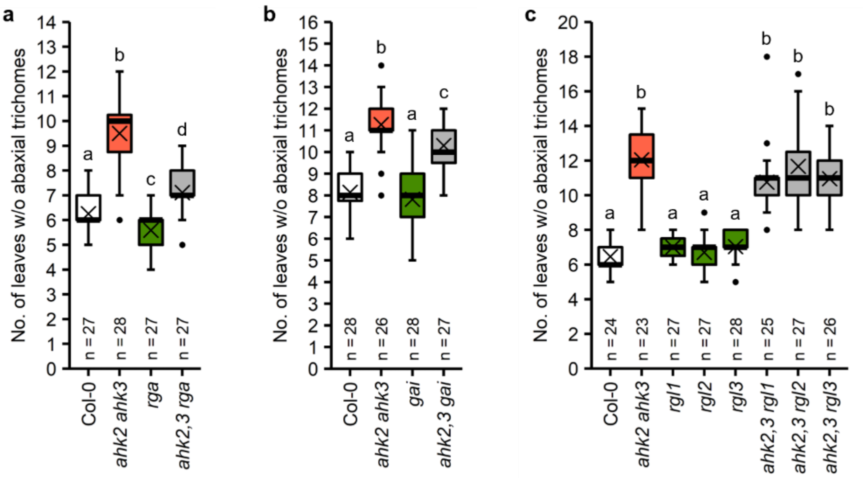

Supplementary Figure 5. Genetic interactions between DELLA genes and CK receptor genes regarding vegetative phase change. Shown are the number of leaves without abaxial trichomes of SD-grown *ahk2,3 rga* (**a**), *ahk2,3 gai* (**b**) and *ahk2,3 rgl* (**c**) hybrid plants. In box plots, the center line represents the median value and the boundaries indicate the 25th percentile (upper) and the 75th percentile (lower). The X marks the mean value. Whiskers extend to the largest and smallest value, excluding outliers which are shown as dots. Letters indicate statistically significant differences between the genotypes, as calculated by Kruskal-Wallis test ($q < 0.05$).

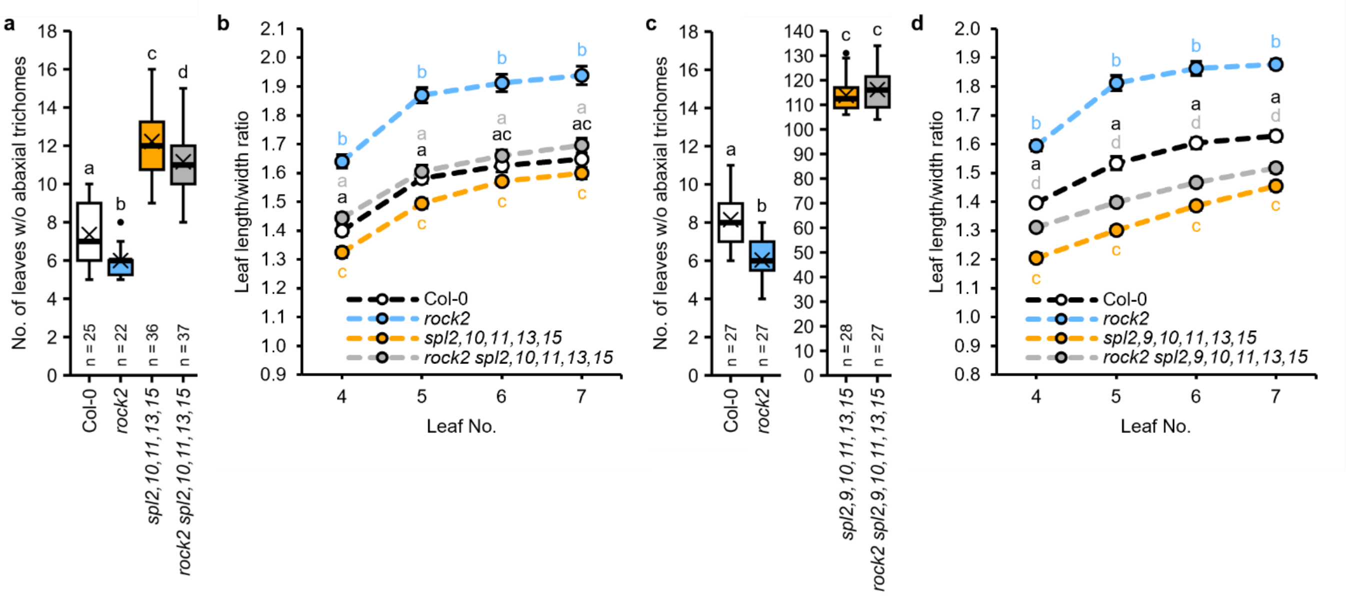

Supplementary Figure 6. SPLs are required for CK to fully exert its influence on VPC. a, c Number of leaves without abaxial trichomes of SD-grown *rock2 spl2,10,11,13,15* (**a**) and *rock2 spl2,9,10,11,13,15* (**c**) hybrid plants in comparison to their respective parents and wild type. In box plots, the center line represents the median value and the boundaries indicate the 25th percentile (upper) and the 75th percentile (lower). The X marks the mean value. Whiskers extend to the largest and smallest value, excluding outliers which are shown as dots. **b, d** Length-to-width ratios of the blades of leaves 4 to 7. Data displayed are expressed as mean ± SEM of SD-grown plants. Numbers of biological replicates: (**b**) Col-0 ($n_4 = 31$; $n_5 = 31$; $n_6 = 31$; $n_7 = 31$), *rock2* ($n_4 = 34$; $n_5 = 34$; $n_6 = 33$; $n_7 = 34$), *spl2,10,11,13,15* ($n_4 = 37$; $n_5 = 36$; $n_6 = 37$; $n_7 = 37$), *rock2 spl2,10,11,13,15* ($n_4 = 36$; $n_5 = 35$; $n_6 = 36$; $n_7 = 36$); (**d**) Col-0 ($n_4 = 36$; $n_5 = 35$; $n_6 = 37$; $n_7 = 38$), *rock2* ($n_4 = 36$; $n_5 = 36$; $n_6 = 35$; $n_7 = 36$), *spl2,9,10,11,13,15* ($n_4 = 38$; $n_5 = 37$; $n_6 = 38$; $n_7 = 38$), *rock2 spl2,9,10,11,13,15* ($n_4 = 35$; $n_5 = 34$; $n_6 = 35$; $n_7 = 35$)). Letters indicate statistically significant differences between the genotypes, as calculated by Kruskal-Wallis test ($q < 0.05$) (**a, c**) or one-way ANOVA, post-hoc Tukey's test ($p < 0.05$) (**b, d**). In case of **b** and **d**, every leaf was analyzed separately.

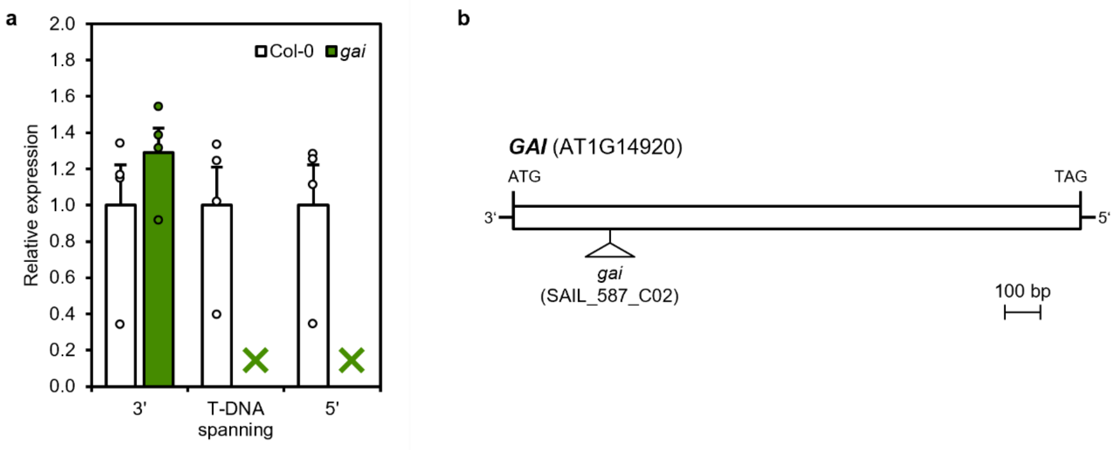

Supplementary Figure 7. Confirmation of *GAI* knockout mutation in the *gai* mutant. a *GAI* transcript levels in LD-grown Col-0 or *gai* seedlings using primer pairs downstream (3') or upstream (5') of the T-DNA insertion or spanning the insertion site. Primers used are listed in Supplementary Table 4. No *GAI* full-length transcript is detectable in the mutant. Transcript levels were determined by qRT-PCR. Data were normalized to *TAFII15* and *PP2AA2*. Data displayed are expressed as mean ± SEM (n = 4 biological replicates). Dots indicate each single biological replicate. **b** Schematic presentation of *GAI* gene structure. It consists of solely one exon (white box), no introns. The T-DNA insertion in the *GAI* gene located at bp 272 is indicated by a triangle.

Supplementary Tables

Supplementary Table 1. Concentrations of GA metabolites in shoots of plants grown under short-day conditions.

	7 DAG, shoot			14 DAG, shoot			21 DAG, shoot		
	Col-0	CKX1ox	*ahk2 ahk3*	Col-0	CKX1ox	*ahk2 ahk3*	Col-0	CKX1ox	*ahk2 ahk3*
	8.71	ND	19.74	ND	ND	11.71	ND	19.54	ND
	13.24	ND	10.68	11.48	5.52	45.84	ND	8.42	ND
	ND	ND	8.37	9.72	ND	16.39	ND	ND	ND
	5.16	ND	ND	14.75	ND	8.91	13.64	19.80	ND
	ND	11.85	ND	9.83	ND	10.15	ND	ND	ND
**GA1**	**9.03**	**11.85**	**12.93**	**11.45**	**5.52**	**18.60**	**13.64**	**15.92**	**-**
SEM	2.34	0.00	3.47	1.17	0.00	6.93	0.00	3.75	-
	14.66	34.11	16.91	22.41	20.54	21.53	22.11	18.53	28.51
	24.73	19.22	28.07	15.78	14.28	25.78	14.62	28.85	23.46
	24.12	10.42	29.86	17.77	11.03	11.27	24.71	27.50	16.69
	17.79	24.50	55.42	16.16	21.31	13.77	15.90	16.29	13.43
	5.61	46.19	27.37	17.26	33.90	22.84	32.72	14.42	24.93
**GA3**	**17.38**	**26.89**	**31.52**	**17.87**	**20.21**	**19.04**	**22.01**	**21.12**	**21.40**
SEM	3.50	6.17	6.39	1.19	3.93	2.78	3.27	2.96	2.77
	167.28	53.98	102.81	130.04	35.50	197.04	76.68	43.38	40.17
	107.03	41.05	141.15	91.11	52.42	211.29	45.40	107.85	28.81
	119.90	60.54	133.04	189.95	42.20	103.77	72.50	50.34	30.74
	149.24	113.95	211.22	177.50	32.53	123.99	49.66	68.44	21.62
	233.89	245.51	81.18	195.15	47.47	107.72	113.01	18.03	18.91
**GA4**	**155.47**	**103.01**	**133.88**	**156.75**	**42.02 ****	**148.76**	**71.45**	**57.61**	**28.05 ****
SEM	22.29	37.73	22.11	20.04	3.68	22.98	12.06	14.94	3.74
	ND	6.33	5.03	2.92	10.76	ND	6.00	ND	7.40
	15.92	6.14	14.45	ND	ND	ND	ND	18.21	11.10
	12.84	14.00	6.14	5.31	ND	ND	5.73	12.80	7.90
	14.30	6.66	10.72	ND	5.53	ND	8.59	14.23	8.15
	13.98	17.56	6.80	10.14	6.44	ND	7.98	7.57	9.31
**GA5**	**14.26**	**10.14**	**8.63**	**6.12**	**7.58**	**-**	**7.08**	**13.20**	**8.77**
SEM	0.64	2.37	1.74	2.12	1.61	-	0.71	2.20	0.66
	355.70	365.26	357.58	197.14	270.37	207.66	198.94	247.18	193.68
	296.02	243.62	329.29	198.91	188.39	253.42	212.36	248.87	175.10
	241.33	338.61	345.31	277.26	174.14	185.98	262.26	168.98	264.06
	281.42	218.77	308.54	211.75	178.45	223.84	176.96	210.04	156.89
	329.80	385.36	256.81	223.43	226.54	301.75	229.11	176.66	181.01
**GA6**	**300.85**	**310.32**	**319.51**	**221.70**	**207.58**	**234.53**	**215.93**	**210.35**	**194.15**
SEM	19.74	33.38	17.69	14.68	18.22	20.09	14.39	16.86	18.46
	ND	ND	ND	ND	ND	ND	ND	ND	ND
	ND	ND	ND	ND	ND	ND	ND	ND	ND
	ND	ND	ND	ND	ND	ND	ND	ND	ND
	ND	ND	ND	ND	ND	ND	ND	ND	ND
	ND	ND	ND	ND	ND	ND	ND	ND	ND
**GA7**	**-**	**-**	**-**	**-**	**-**	**-**	**-**	**-**	**-**
SEM	-	-	-	-	-	-	-	-	-
	36.39	37.01	72.55	44.82	42.48	36.09	27.23	6.84	17.26
	33.03	39.14	72.75	53.11	30.68	47.55	31.29	22.46	37.45
	39.33	34.45	58.73	75.98	23.81	29.84	29.51	8.43	26.96
	22.00	47.87	82.71	49.14	34.87	30.97	19.54	7.93	15.97
	32.75	59.73	49.72	39.50	27.92	41.10	18.39	10.20	18.49
**GA8**	**32.70**	**43.64**	**67.29 ****	**52.51**	**31.95 ****	**37.11 ***	**25.19**	**11.17 ***	**23.23**
SEM	2.93	4.61	5.82	6.29	3.19	3.29	2.63	2.87	4.04
	18.11	9.83	ND	5.34	29.23	ND	ND	6.02	5.92
	9.87	10.86	12.58	ND	ND	ND	ND	22.38	ND
	5.57	15.71	5.98	5.13	ND	6.94	ND	ND	ND
	7.74	ND	83.25	9.97	ND	7.35	13.50	15.35	ND
	18.39	6.60	24.58	17.41	ND	17.20	17.63	5.45	ND
**GA9**	**11.94**	**10.75**	**31.60**	**9.46**	**29.23**	**10.50**	**15.57**	**12.30**	**5.92**
SEM	2.67	1.89	17.64	2.88	0.00	3.35	2.06	4.05	0.00

Supplementary Table 1. Continuation.

	7 DAG, shoot			14 DAG, shoot			21 DAG, shoot		
	**Col-0**	**CKX1ox**	***ahk2 ahk3***	**Col-0**	**CKX1ox**	***ahk2 ahk3***	**Col-0**	**CKX1ox**	***ahk2 ahk3***
	ND	ND	ND	ND	ND	ND	ND	ND	ND
	ND	ND	ND	ND	ND	ND	ND	ND	ND
	ND	ND	ND	ND	ND	ND	ND	ND	ND
	ND	ND	ND	ND	ND	ND	ND	ND	ND
	ND	ND	ND	ND	ND	ND	ND	ND	ND
**GA12**	-	-	-	-	-	-	-	-	-
SEM	-	-	-	-	-	-	-	-	-
	88.03	35.73	46.73	30.37	386.95	156.07	229.22	186.04	181.85
	49.68	30.67	31.96	28.02	299.70	204.80	202.99	175.12	100.74
	34.00	28.32	66.47	42.18	235.79	162.37	254.58	137.49	208.06
	45.34	17.15	47.03	18.37	155.44	133.66	146.33	152.28	72.31
	76.41	57.84	26.15	27.08	188.42	195.78	165.44	159.42	146.81
**GA13**	**58.69**	**33.94**	**43.67**	**29.21**	**253.26 ****	**170.54 ***	**199.71**	**162.07**	**141.96**
SEM	10.11	6.70	7.02	3.83	41.30	13.13	19.90	8.52	25.03
	ND	ND	ND	ND	ND	ND	ND	ND	ND
	ND	ND	ND	ND	ND	ND	ND	ND	ND
	ND	ND	ND	ND	ND	ND	ND	ND	ND
	ND	ND	ND	ND	ND	ND	ND	ND	ND
	ND	ND	ND	ND	ND	ND	ND	ND	ND
**GA15**	-	-	-	-	-	-	-	-	-
SEM	-	-	-	-	-	-	-	-	-
	47.83	35.22	37.29	108.20	73.28	142.56	70.11	38.58	91.60
	55.99	25.48	57.11	125.04	56.05	141.48	76.72	76.68	107.08
	56.02	24.77	41.93	123.72	81.90	116.20	122.44	30.88	100.45
	29.01	45.94	53.04	119.79	59.79	108.06	100.42	52.33	89.31
	30.27	54.58	55.81	97.82	68.57	176.91	64.91	55.35	70.04
**GA19**	**43.82**	**37.20**	**49.04**	**114.91**	**67.92 ***	**137.04**	**86.92**	**50.77**	**91.70**
SEM	5.98	5.81	3.97	5.20	4.65	12.07	10.76	7.87	6.28
	334.44	590.55	599.43	111.38	225.24	126.75	207.47	140.69	127.68
	190.22	328.65	488.05	161.04	139.13	111.83	60.45	115.17	119.80
	239.54	300.13	392.64	137.73	135.24	293.79	149.04	78.15	97.25
	297.50	239.84	492.23	95.02	100.56	272.38	78.14	109.33	140.05
	188.30	546.33	328.25	87.64	242.59	299.10	63.28	131.48	133.97
**GA20**	**250.00**	**401.10 ***	**460.12 ***	**118.56**	**168.55**	**220.77**	**111.68**	**114.96**	**123.75**
SEM	29.05	70.15	46.46	13.66	27.65	41.74	28.86	10.77	7.43
	5.22	11.29	ND	6.05	ND	ND	ND	ND	30.33
	5.58	ND	ND	15.11	8.08	6.68	8.38	18.23	11.55
	ND	ND	ND	6.01	7.87	5.20	16.24	ND	254.58
	8.11	ND	14.67	10.81	ND	9.22	-	ND	146.33
	ND	7.18	29.75	ND	ND	ND	39.11	9.11	165.44
**GA24**	**6.31**	**9.24**	**22.21**	**9.49**	**7.98**	**7.03**	**21.24**	**13.67**	**121.64**
SEM	0.91	2.06	7.54	2.18	0.10	1.17	9.22	4.56	45.09
	3.90	100.91	46.04	5.76	48.24	19.35	ND	11.68	28.43
	16.07	31.15	51.41	ND	25.94	19.09	9.01	13.71	14.11
	15.86	44.10	42.99	20.00	25.30	5.80	ND	14.30	ND
	21.32	28.79	54.26	17.48	6.20	5.55	18.93	11.46	8.20
	53.76	48.50	50.70	14.84	9.35	20.79	7.31	15.32	10.31
**GA29**	**22.18**	**50.69**	**49.08**	**14.52**	**23.01**	**14.12**	**11.75**	**13.29**	**15.26**
SEM	8.39	13.10	2.01	3.10	7.48	3.46	3.62	0.75	4.56
	278.49	18.51	160.29	222.39	38.77	97.40	39.49	10.98	18.45
	238.00	43.22	168.53	279.66	30.79	96.29	41.24	8.20	17.51
	262.72	47.58	160.20	316.46	33.40	96.62	51.11	9.61	17.17
	238.55	57.07	94.14	187.61	34.45	116.40	51.69	11.80	14.71
	169.74	58.14	117.86	297.91	32.82	127.78	39.76	7.46	19.99
**GA34**	**237.50**	**44.90 *****	**140.20 ***	**260.81**	**34.05 *****	**106.90 ***	**44.66**	**9.61 *****	**17.56 ***
SEM	18.59	7.18	14.54	24.15	1.32	6.46	2.77	0.81	0.87

Supplementary Table 1. Continuation.

	7 DAG, shoot			14 DAG, shoot			21 DAG, shoot		
	**Col-0**	**CKX1ox**	***ahk2 ahk3***	**Col-0**	**CKX1ox**	***ahk2 ahk3***	**Col-0**	**CKX1ox**	***ahk2 ahk3***
	17.16	74.85	38.66	38.56	34.44	21.97	20.33	19.28	48.14
	12.33	23.83	48.96	43.06	15.47	57.28	26.70	98.36	31.03
	7.59	48.93	48.86	83.06	10.88	52.81	33.81	30.89	56.07
	14.27	19.72	28.82	39.19	25.67	82.50	50.97	43.04	58.19
	26.87	38.62	101.47	56.71	41.94	119.26	24.31	66.76	43.46
**GA44**	**15.64**	**41.19 ***	**53.35 ****	**52.12**	**25.68**	**66.76**	**31.22**	**51.67**	**47.38**
SEM	3.21	9.90	12.59	8.40	5.76	16.27	5.40	14.08	4.88
	136.09	32.52	90.11	43.44	75.55	70.73	12.09	347.72	268.47
	200.78	50.75	46.06	38.84	81.07	59.76	20.08	322.57	335.08
	142.84	14.78	73.59	41.87	37.54	135.33	6.61	164.55	365.87
	59.94	92.59	28.17	225.89	75.90	119.82	16.91	118.58	316.36
	54.91	49.21	38.45	23.56	62.79	35.61	33.45	122.82	215.04
**GA51**	**118.91**	**47.97**	**55.27**	**74.72**	**66.57**	**84.25**	**17.83**	**215.25 ***	**300.16 ****
SEM	27.52	12.92	11.51	37.96	7.86	18.74	4.52	49.76	26.50
	12.31	14.51	10.60	21.27	7.38	13.95	10.51	3.82	33.91
	6.02	8.99	33.86	48.94	4.73	14.12	30.08	7.61	26.41
	2.67	8.78	5.12	30.19	11.48	45.20	28.74	4.96	20.37
	1.87	0.80	4.61	22.56	7.41	23.26	178.40	7.29	18.19
	7.29	9.63	7.44	29.77	4.77	23.40	52.68	4.88	11.85
**GA53**	**6.03**	**8.54**	**12.32**	**30.55**	**7.16 ****	**23.98**	**60.08**	**5.71 ****	**22.15**
SEM	1.87	2.20	5.49	4.94	1.23	5.70	30.33	0.74	3.75

Mean of 5 biological replicates is shown in bolt type. Values are given as fmol x (g fresh weight)$^{-1}$. Asterisks indicate statistically significant differences compared to the wild type of the respective time point, as calculated by Kruskal-Wallis test (*$q < 0.05$; **$q < 0.01$; ***$q < 0.001$). ND = not detected.

Supplementary Table 2. Mutants and transgenic lines used in this study.

Genotype	Named in this study	Reference
arr1-3 arr10-5 arr12-1	*arr1,10,12*	Mason et al., 2005[1]
ahk2-2tk ahk3-3	*ahk2 ahk3*	Higuchi et al., 2004[2]
rock2	*rock2*	Bartrina et al., 2017[3]
p35S:CKX1	CKX1ox	Werner et al., 2003[4]
ga1	*ga1*	Willige et al., 2011[5]
rock2 ga1	*rock2 ga1*	this study
ga3ox1-3 ga3ox2-1	*ga3ox1,2*	Mitchum et al., 2006[6]
rock2 ga3ox1-3 ga3ox2-1	*rock2 ga3ox1,2*	this study
gid1b-1 gid1c-2	*gid1b gid1c*	Griffiths et al., 2006[7]
rock2 gid1b-1 gid1c-2	*rock2 gid1b,c*	this study
gai	*gai*	Matschi et al., 2015[8]
ahk2-2tk ahk3-3 gai	*ahk2,3 gai*	this study
rga-28	*rga*	Tyler et al., 2004[9]
ahk2-2tk ahk3-3 rga-28	*ahk2,3 rga*	this study
gai rga-28	*gai rga*	this study
ahk2-2tk ahk3-3 gai rga-28	*ahk2,3 gai rga*	this study
spl2-1 spl10-2 spl11-1 spl13-1 spl15-1	*spl2,10,11,13,15*	this study
rock2 spl2-1 spl10-2 spl11-1 spl13-1 spl15-1	*rock2 spl2,10,11,13,15*	this study
spl2-1 spl9-4 spl10-2 spl11-1 spl13-1 spl15-1	*spl2,9,10,11,13,15*	this study
rock2 spl2-1 spl9-4 spl10-2 spl11-1 spl13-1 spl15-1	*rock2 spl2,9,10,11,13,15*	this study
toe1-2 toe2-1	*toe1 toe2*	Aukerman & Sakai, 2003[10]
ahk2-2tk ahk3-3 toe1-2 toe2-1	*ahk2,3 toe1,2*	Werner et al., 2021[11]
gai rga-28 toe1-2 toe2-1	*gai rga toe1,2*	this study
ahk2-2tk ahk3-3 gai rga-28 toe1-2 toe2-1	*ahk2,3 gai rga toe1,2*	this study
rgl1-2	*rgl1*	Tyler et al., 2004[9]
ahk2-2tk ahk3-3 rgl1-2	*ahk2,3 rgl1*	this study
rgl2-13	*rgl2*	Tyler et al., 2004[9]
ahk2-2tk ahk3-3 rgl2-13	*ahk2,3 rgl2*	this study
rgl3-3	*rgl3*	Tyler et al., 2004[9]
ahk2-2tk ahk3-3 rgl3-3	*ahk2,3 rgl3*	this study

Supplementary Table 3. Gene-specific and T-DNA primers used for genotyping in this study.

Locus	Allele	Primer pair	Sequences (5' → 3')	Fragment size
AHK2 (AT5G35750)	WT *(ahk2-2tk)*	446_AHK2-ahk2-2tk-2_fw<br>447_AHK2-ahk2-2tk-2_rv	TGCCTTGTCTATTCTTGTATCT<br>TAGGTTCAATTTCTTCAGTCC	719 bp
	ahk2-2tk	446_AHK2-ahk2-2tk-2_fw<br>230_T-DNA-ahk2-2tk_rv	TGCCTTGTCTATTCTTGTATCT<br>ATAACGCTGCGGACATCTAC	~ 700 bp
AHK3 (AT1G27320)	WT *(ahk3-3)*	397_AHK3-ahk3-3_fw<br>449_AHK3-ahk3-3_rv	GCAAGAATCCAGGTGCTAAC<br>GCTATCAGTTACAACCCTTGC	771 bp
	ahk3-3	315_LBa1<br>449_AHK3-ahk3-3_rv	TGGTTCACGTAGTGGGCCATCG<br>GCTATCAGTTACAACCCTTGC	~ 850 bp
ARR1 (AT3G16857)	WT	309_ARR1-arr1-3_fw<br>310_ARR1-arr1-3_rv	CTTCAAGCACTAGCCGTCACAGGTCAGTT<br>AATGTTATCGATGGAGTATGCGTCAAAGT	1306 bp
	arr1-3	309_ARR1-arr1-3_fw<br>315_LBa1	CTTCAAGCACTAGCCGTCACAGGTCAGTT<br>TGGTTCACGTAGTGGGCCATCG	953 bp
ARR10 (AT4G31920)	WT	311_ARR10-arr10-5_fw<br>312_ARR10-arr10-5_rv	CATTGGAGTTGTTGAGGGAGA<br>CGATGATGAGACTGGTTGGA	1075 bp
	arr10-5	311_ARR10-arr10-5_fw<br>315_LBa1	CATTGGAGTTGTTGAGGGAGA<br>TGGTTCACGTAGTGGGCCATCG	1230 bp
ARR12 (AT2G25180)	WT	313_ARR12-arr12-1_fw<br>314_ARR12-arr12-1_rv	TAACAACGACGAACCAAGCA<br>TTGGCAGAGTCACAGAATGG	1654 bp
	arr12-1	313_ARR12-arr12-1_fw<br>315_LBa1	TAACAACGACGAACCAAGCA<br>TGGTTCACGTAGTGGGCCATCG	962 bp
GA1 (AT4G02780)	WT	689_GA1-ga1_fw<br>691_GA1-ga1_rv	CGAGTAACCACTTCTCCTGT<br>CTAACGCAAACCCAATCTATC	962 bp
	ga1-3	689_GA1-ga1_fw<br>690_T-DNA-ga1_rv	CGAGTAACCACTTCTCCTGT<br>GCTGTTGCCCGTCTCAC	704 bp
GA3OX1 (AT1G15550)	WT	696_GA3OX1-ga3ox1-3_fw<br>697_GA3OX1-ga3ox1-3_rv	GTGTTTAGAGGCCATCCCATTCAC<br>CAAACAAATCATATTGCTGAAATC	1569 bp
	ga3ox1-3	696_GA3OX1-ga3ox1-3_fw<br>676_JMLB1	GTGTTTAGAGGCCATCCCATTCAC<br>GGCAATCAGCTGTTGCCCGTCTCACTGGTG	~ 1500 bp
GA3OX2 (AT1G80340)	WT	698_GA3OX2-ga3ox2-1_fw<br>699_GA3OX2-ga3ox2-1_rv	GCCTTTTAGCATGAGTTCAAC<br>AGATCATTATATCGGATGGTG	1357 bp
	ga3ox2-1	698_GA3OX2-ga3ox2-1_fw<br>315_LBa1	GCCTTTTAGCATGAGTTCAAC<br>TGGTTCACGTAGTGGGCCATCG	~ 1800 bp
GID1B (AT3G63010)	WT	653_GID1B-gid1b-1_fw<br>654_GID1B-gid1b-1_rv	TCTCCTGTCCACCAAACATTG<br>CTGGGTTTTGGAGACTATGGC	897 bp
	gid1b-1	655_SLAT-3'<br>654_GID1B-gid1b-1_rv	CTTATTTCAGTAAGAGTGTGGGGTTTTGG<br>CTGGGTTTTGGAGACTATGGC	354 bp

Supplementary Table 3. Continuation.

Locus	Allele	Primer pair	Sequences (5' → 3')	Fragment size
GID1C (AT5G27320)	WT	656_GID1C-gid1c-2_fw<br>657_GID1C-gid1c-2_rv	TCACCTTCCATGGTGGATATC<br>CTTTTAACCGTCATCTCGCAG	983 bp
	gid1c-2	658_GABIKatLB1<br>657_GID1C-gid1c-2_rv	CCCATTTGGACGTCAATGTAGACAC<br>CTTTTAACCGTCATCTCGCAG	671 bp
GAI (AT1G14920)	WT	661_GAI-gai_fw<br>663_GAI-gai_2_rv	ATGAAGAGAGATCATCATCAT<br>AGCTTCGGCGAAGTAAGTAGC	648 bp
	gai	401_LB1-SAIL<br>662_GAI-gai_rv	GCCTTTTCAGAAATGGATAAATAGCCTTGCTTCC<br>ACCTTATCGATCGCACCAGGT	1064 bp
RGA (AT2G01570)	WT	664_RGA-rga-28_2_fw<br>665_RGA-rga-28_2_rv	TCACATAGAGAAGTCACATG<br>CAGCTAAGCATCCGATTTGC	882 bp
	rga-28	660_RGA-rga-28_rv<br>400_LBb1.3-SALK	TGGTTCGCCGTGAAGTG<br>ATTTTGCCGATTTCGGAAC	~ 900 bp
RGL1 (AT1G66350)	WT	667_RGL1-rgl1-2_fw<br>668_RGL1-rgl1-2_rv	GATATTCAAGAAGTTGGTTGG<br>ACATTATACCCATCAGCCC	566 bp
	rgl1-2	667_RGL1-rgl1-2_fw<br>675_JMRB2	GATATTCAAGAAGTTGGTTGG<br>TGATAGTGACCTTAGGCGACTTTTGAACGC	~ 700 bp
RGL2 (AT3G03450)	WT	669_RGL2-rgl2-13_fw<br>670_RGL2-rgl2-13_rv	GTGGTGCTCGTTGACTCTC<br>AACTCGGTCTTGACTCGG	861 bp
	rgl2-13	669_RGL2-rgl2-13_fw<br>315_LBa1	GTGGTGCTCGTTGACTCTC<br>TGGTTCACGTAGTGGGCCATCG	~ 900 bp
RGL3 (AT5G17490)	WT	671_RGL3-rgl3-3_fw<br>672_RGL3-rgl3-3_rv	CGAAGCCATCAAGAAACG<br>GTGAGACGAAACGACGGT	869 bp
	rgl3-3	671_RGL3-rgl3-3_fw<br>676_JMLB1	CGAAGCCATCAAGAAACG<br>GGCAATCAGCTGTTGCCCGTCTCACTGGTG	~ 500 bp
SPL2 (AT5G43270)	WT	735_SPL2-spl2-1_fw<br>736_SPL2-spl2-1_rv	TGAATAGTGGAAGAGAGAAAGCTTC<br>CTTTAAACCGAGAACCGGATC	983 bp
	spl2-1	735_SPL2-spl2-1_fw<br>315_LBa1	TGAATAGTGGAAGAGAGAAAGCTTC<br>TGGTTCACGTAGTGGGCCATCG	~ 700 bp
SPL9 (AT2G42200)	WT	420_SPL9-spl9-4_fw<br>421_SPL9-spl9-4_rv	TGGTTCCTCCACTGAGTCATC<br>GCTCATTATGACCAGCGAGTC	1021 bp
	spl9-4	401_LB1-SAIL<br>421_SPL9-spl9-4_rv	GCCTTTTCAGAAATGGATAAATAGCCTTGCTTCC<br>GCTCATTATGACCAGCGAGTC	~ 650 bp
SPL10 (AT1G27370)	WT	746_SPL10-spl10-2_fw<br>747_SPL10-spl10-2_rv	AGGACAAACGATGCAATCTTG<br>TTTTCTTCCGAGCAACAACAG	240 bp
	spl10-2	746_SPL10-spl10-2_fw<br>747_SPL10-spl10-2_rv	AGGACAAACGATGCAATCTTG<br>TTTTCTTCCGAGCAACAACAG	205 bp

Supplementary Table 3. Continuation.

Locus	Allele	Primer pair	Sequences (5' → 3')	Fragment size
SPL11 (AT1G27360)	WT	741_SPL11-spl11-1_fw<br>742_SPL11-spl11-1_rv	GTTGCATTCTCTTTAGATTTTACTGTA<br>GGACGAGGTTTTTATCATAGGTTTGG	955 bp
	spl11-1	741_SPL11-spl11-1_fw<br>743_T-DNA-spl11-1	GTTGCATTCTCTTTAGATTTTACTGTA<br>TGTGCCAGGTGCCCACGGAATAGT	~ 200 bp
SPL13A/B (AT5G50570/ AT5G50670)	WT	748_SPL13-spl13-1_fw<br>749_SPL13-spl13-1_rv	TTCAAAGAGAACAAGAGGGA<br>CTTAAACCATTAACACACAATCT	215 bp<br>not cleaved by HpaI
	spl13-1	748_SPL13-spl13-1_fw<br>749_SPL13-spl13-1_rv	TTCAAAGAGAACAAGAGGGA<br>CTTAAACCATTAACACACAATCT	176 + 39 bp<br>cleaved by HpaI
SPL15 (AT3G57920)	WT	422_SPL15-spl15-1_fw<br>423_SPL15-spl15-1_rv	TCCACCGAGTCTTCTTCACTC<br>TGTTGGTGTCTGAAGTTGCTG	1013 bp
	spl15-1	400_LBb1.3-SALK<br>423_SPL15-spl15-1_rv	ATTTTGCCGATTTCGGAAC<br>TGTTGGTGTCTGAAGTTGCTG	~ 800 bp
TOE1 (AT2G28550)	WT	404_TOE1-toe1-2_fw<br>405_TOE1-toe1-2_rv	GAAGAGTTTGTGCATATACTGCG<br>GAAGGGAAGTGAAAGAGCCTC	569 bp
	toe1-2	400_LBb1.3-SALK<br>405_TOE1-toe1-2_rv	ATTTTGCCGATTTCGGAAC<br>GAAGGGAAGTGAAAGAGCCTC	~ 400 bp
TOE2 (AT5G60120)	WT	406_TOE2-toe2-1_fw<br>407_TOE2-toe2-1_rv	AGTTGTGCTCTACACGAACGG<br>TCCCAGCAGAAATCAGTTCAC	580 bp
	toe2-1	406_TOE2-toe2-1_fw<br>400_LBb1.3-SALK	AGTTGTGCTCTACACGAACGG<br>ATTTTGCCGATTTCGGAAC	~ 350 bp
ROCK2	WT	263_rock2_fw<br>264_rock2_rv	TGGCTCAGAAATTGGGGATA<br>TGATGGCTTCATATAAAATATAACCATCTA	250 + 31 bp<br>cleaved by XbaI
	rock2	263_rock2_fw<br>264_rock2_rv	TGGCTCAGAAATTGGGGATA<br>TGATGGCTTCATATAAAATATAACCATCTA	281 bp<br>not cleaved by XbaI

Supplementary Table 4. qRT primers used for detection of gene transcripts in this study.

Gene	Primers	Sequences (5' → 3')
PP2AA2 (AT3G25800)	478_PP2AA2-qRT_fw	CCATTAGATCTTGTCTCTCTGCT
	479_PP2AA2-qRT_rv	GACAAAACCCGTACCGAG
TAFII15 (AT4G31720)	339_TAFII15-qRT_fw	gaatcacggccaacaatc
	340_TAFII15-qRT_rv	actcttagccaagtagtgctcc
GA1 (AT4G02780)	842_GA1-qRT6_fw	CCTAACTAACTTCTTCACCCACAC
	843_GA1-qRT6_rv	AGGAAGACAGATTCGATTGATG
GA2 (AT1G79460)	785_GA2-qRT_fw	TGAGCACTATGGGTCGTCTTC
	786_GA2-qRT_rv	ACCACACTTCCTTTCTCCTCC
GA3 (AT5G25900)	787_GA3-qRT_fw	CCCATTCGCTACGCCCA
	788_GA3-qRT_rv	TCTCCCAACGCTTCTTATCCA
KAO1 (AT1G05160)	789_KAO1-qRT_fw	GTGCGTTCCTTCCTTTTGGT
	790_KAO1-qRT_rv	ATCACTGGACATTCGGGGTT
KAO2 (AT2G32440)	791_KAO2-qRT_fw	GAAATGGGTGCTGAAGAGAGTG
	792_KAO2-qRT_rv	GGTGATGTAGGATTGGATGAAGG
GA20ox1 (AT4G25420)	812_GA20ox1-qRT_fw	GCTACGCAAGCAGTTTCACC
	813_GA20ox1-qRT_rv	ATGTCCCAACGCATCGCA
GA20ox2 (AT5G51810)	814_GA20ox2-qRT_fw	AGAGCGGTTGTGAATAGAGAGAG
	815_GA20ox2-qRT_rv	TCAAGGAACATAGACCAAGTGAAG
GA3ox1 (AT1G15550)	701_GA3ox1-qRT_fw	TTGGGGTCAGCGAAGAAGA
	702_GA3ox1-qRT_rv	ACAGAATGGTTAGGAGGGTGG
GA3ox2 (AT1G80340)	893_GA3ox2-qRT5_6_fw	GCCACCACCTCAAATACTGTG
	894_GA3ox2-qRT5_rv	ATGAGGGTCGAGTCTGTATGG
GA2ox1 (AT1G78440)	872_GA2ox1-qRT2_fw	AACGTTGGTGACTCTCTCCAGGTG
	873_GA2ox1-qRT2_rv	AACCCTATGCCTCACGCTCTTG
GA2ox2 (AT1G30040)	874_GA2ox2-qRT2_fw	AGATGGAAGTTGGGTCGCTGTC
	875_GA2ox2-qRT2_rv	CCCGTTAGTCATAACCTGAAGAGC
GAI 5' end (AT1G14920)	683_GAI-5'-qRT_fw	TGAATGAAGAAGACGACGGTAA
	684_GAI-5'-qRT_rv	CGGTGAGCATAGAATCAAGCC
GAI T-DNA spanning (AT1G14920)	687_GAI-T-DNA-qRT_fw	CTTGATTCTATGCTCACCGACC
	688_GAI-T-DNA-qRT_rv	CACCGTTCTCCTGCGAGT
GAI 3' end (AT1G14920)	685_GAI-3'-qRT_fw	TTCGGCTTCTTCGTCTAACC
	686_GAI-3'-qRT_rv	TGCGAGTCAACCAGGACAA

Supplementary References

1. Mason, M. G., et al. Multiple type-B response regulators mediate cytokinin signal transduction in *Arabidopsis*. *Plant Cell* **17**, 3007-3018 (2005).
2. Higuchi, M., et al. *In planta* functions of the *Arabidopsis* cytokinin receptor family. *Proc. Natl. Acad. Sci. USA* **101**, 8821-8826 (2004).
3. Bartrina, I., Jensen, H., Novák, O., Strnad, M., Werner, T. & Schmülling, T. Gain-of-function mutants of the cytokinin receptors AHK2 and AHK3 regulate plant organ size, flowering time and plant longevity. *Plant Physiology* **173**, 1783-1797 (2017).
4. Werner, T., Motyka, V., Laucou, V., Smets, R., Van Onckelen, H. & Schmülling, T. Cytokinin-deficient transgenic *Arabidopsis* plants show multiple developmental alterations indicating opposite functions of cytokinins in the regulation of shoot and root meristem activity. *Plant Cell* **15**, 2532-2550 (2003).
5. Willige, B. C., Isono, E., Richter, R., Zourelidou, M. & Schwechheimer, C. Gibberellin regulates PIN-FORMED abundance and is required for auxin transport-dependent growth and development in *Arabidopsis thaliana*. *Plant Cell* **23**, 2184-2195 (2011).
6. Mitchum, M. G., et al. Distinct and overlapping roles of two gibberellin 3-oxidases in *Arabidopsis* development. *Plant Journal* **45**, 804-818 (2006).
7. Griffiths, J., et al. Genetic characterization and functional analysis of the GID1 gibberellin receptors in *Arabidopsis*. *Plant Cell* **18**, 3399-3414 (2006).
8. Matschi, S., Hake, K., Herde, M., Hause, B. & Romeis, T. The calcium-dependent protein kinase CPK28 regulates development by inducing growth phase-specific, spatially restricted alterations in jasmonic acid levels independent of defense responses in *Arabidopsis*. *Plant Cell* **27**, 591-606 (2015).
9. Tyler, L., et al. DELLA proteins and gibberellin-regulated seed germination and floral development in *Arabidopsis*. *Plant Physiology* **135**, 1008-1019 (2004).
10. Aukerman, M. J. & Sakai, H. Regulation of flowering time and floral organ identity by a microRNA and its *APETALA2*-like target genes. *Plant Cell* **15**, 2730-2741 (2003).
11. Werner, S., Bartrina, I. & Schmülling, T. Cytokinin regulates vegetative phase change in *Arabidopsis thaliana* through the miR172/TOE1-TOE2 module. *Nat. Commun.* **12**, 5816 (2021).
