# Supplementary material for: Cytokinin depends on GA biosynthesis and signaling to regulate different aspects of vegetative phase change in Arabidopsis
Source: Nat Commun. 2025 Jul 8;16:6292. doi: 10.1038/s41467-025-61507-5 (PMC12238502; doi:10.1038/s41467-025-61507-5)
Supplement: Supplementary file 2 — Reporting Summary [file 41467_2025_61507_MOESM2_ESM.pdf]

nature portfolio

Corresponding author(s): Thomas Schmülling

Last updated by author(s): Jun 16, 2025

Reporting Summary

Nature Portfolio wishes to improve the reproducibility of the work that we publish. This form provides structure for consistency and transparency in reporting. For further information on Nature Portfolio policies, see our Editorial Policies and the Editorial Policy Checklist.

Statistics

For all statistical analyses, confirm that the following items are present in the figure legend, table legend, main text, or Methods section.

n/a	Confirmed	
☐	☒	The exact sample size (*n*) for each experimental group/condition, given as a discrete number and unit of measurement
☐	☒	A statement on whether measurements were taken from distinct samples or whether the same sample was measured repeatedly
☐	☒	The statistical test(s) used AND whether they are one- or two-sided<br>*Only common tests should be described solely by name; describe more complex techniques in the Methods section.*
☒	☐	A description of all covariates tested
☐	☒	A description of any assumptions or corrections, such as tests of normality and adjustment for multiple comparisons
☐	☒	A full description of the statistical parameters including central tendency (e.g. means) or other basic estimates (e.g. regression coefficient) AND variation (e.g. standard deviation) or associated estimates of uncertainty (e.g. confidence intervals)
☐	☒	For null hypothesis testing, the test statistic (e.g. *F*, *t*, *r*) with confidence intervals, effect sizes, degrees of freedom and *P* value noted<br>*Give P values as exact values whenever suitable.*
☒	☐	For Bayesian analysis, information on the choice of priors and Markov chain Monte Carlo settings
☒	☐	For hierarchical and complex designs, identification of the appropriate level for tests and full reporting of outcomes
☒	☐	Estimates of effect sizes (e.g. Cohen's *d*, Pearson's *r*), indicating how they were calculated

Our web collection on statistics for biologists contains articles on many of the points above.

Software and code

Policy information about availability of computer code

Data collection	qRT-PCR experiments were performed using Bio-Rad CFX96 Real-Time System and Bio-Rad CFX Manager (v3.1.1517.0823). For leaf shape analysis, leaf images were measured using ImageJ (v1.54p).
Data analysis	For statistical analysis, Prism v9 (GraphPad) was used. Box plots were generated using RStudio (v2024.12.1+563) with R (v4.4.3) and the ggplot2 (version 3.5.1) package. Bar charts were generated using Microsoft Excel Professional Plus 2021. qRT-PCR data analysis was carried out according to Vandesompele et al. (2002).

For manuscripts utilizing custom algorithms or software that are central to the research but not yet described in published literature, software must be made available to editors and reviewers. We strongly encourage code deposition in a community repository (e.g. GitHub). See the Nature Portfolio guidelines for submitting code & software for further information.

Data

Policy information about availability of data

All manuscripts must include a data availability statement. This statement should provide the following information, where applicable:

- Accession codes, unique identifiers, or web links for publicly available datasets
- A description of any restrictions on data availability
- For clinical datasets or third party data, please ensure that the statement adheres to our policy

Source data and statistical information are provided with this paper.

Research involving human participants, their data, or biological material

Policy information about studies with human participants or human data. See also policy information about sex, gender (identity/presentation), and sexual orientation and race, ethnicity and racism.

Reporting on sex and gender	*Use the terms sex (biological attribute) and gender (shaped by social and cultural circumstances) carefully in order to avoid confusing both terms. Indicate if findings apply to only one sex or gender; describe whether sex and gender were considered in study design; whether sex and/or gender was determined based on self-reporting or assigned and methods used. Provide in the source data disaggregated sex and gender data, where this information has been collected, and if consent has been obtained for sharing of individual-level data; provide overall numbers in this Reporting Summary. Please state if this information has not been collected. Report sex- and gender-based analyses where performed, justify reasons for lack of sex- and gender-based analysis.*
Reporting on race, ethnicity, or other socially relevant groupings	*Please specify the socially constructed or socially relevant categorization variable(s) used in your manuscript and explain why they were used. Please note that such variables should not be used as proxies for other socially constructed/relevant variables (for example, race or ethnicity should not be used as a proxy for socioeconomic status). Provide clear definitions of the relevant terms used, how they were provided (by the participants/respondents, the researchers, or third parties), and the method(s) used to classify people into the different categories (e.g. self-report, census or administrative data, social media data, etc.) Please provide details about how you controlled for confounding variables in your analyses.*
Population characteristics	*Describe the covariate-relevant population characteristics of the human research participants (e.g. age, genotypic information, past and current diagnosis and treatment categories). If you filled out the behavioural & social sciences study design questions and have nothing to add here, write "See above."*
Recruitment	*Describe how participants were recruited. Outline any potential self-selection bias or other biases that may be present and how these are likely to impact results.*
Ethics oversight	*Identify the organization(s) that approved the study protocol.*

Note that full information on the approval of the study protocol must also be provided in the manuscript.

Field-specific reporting

Please select the one below that is the best fit for your research. If you are not sure, read the appropriate sections before making your selection.

☒ Life sciences ☐ Behavioural & social sciences ☐ Ecological, evolutionary & environmental sciences

For a reference copy of the document with all sections, see nature.com/documents/nr-reporting-summary-flat.pdf

Life sciences study design

All studies must disclose on these points even when the disclosure is negative.

Sample size	No statistical method was used to predetermine sample size. The sample sizes were chosen according to the standard in the field of research. They are standard for this type of experiments and the reproducibility between independent experiments indicates sample sizes to be sufficient.
Data exclusions	No data were purposely excluded. However, individual plants that died or developed abnormal phenotypes during the experiments were not examined and therefore excluded from the analysis. Single samples during qRT-PCR analyses did not yield a fluorescence signal, but respective runs were not repeated if other samples were fine.
Replication	All experiments were repeated at least two times with similar results, with the exception of GA metabolite measurements, which were only performed once.
Randomization	Samples were grown randomly placed within the same growth chamber to avoid positional effects, and allocated to groups based on genotype and treatment.
Blinding	The researchers were not blinded during this study, which would have been difficult due to the obvious phenotypes of the genetic material. Most of the results are quantitative, or not subjective.

Reporting for specific materials, systems and methods

We require information from authors about some types of materials, experimental systems and methods used in many studies. Here, indicate whether each material, system or method listed is relevant to your study. If you are not sure if a list item applies to your research, read the appropriate section before selecting a response.

Materials & experimental systems

n/a	Involved in the study
☒	☐ Antibodies
☒	☐ Eukaryotic cell lines
☒	☐ Palaeontology and archaeology
☒	☐ Animals and other organisms
☒	☐ Clinical data
☒	☐ Dual use research of concern
☐	☒ Plants

Methods

n/a	Involved in the study
☒	☐ ChIP-seq
☒	☐ Flow cytometry
☒	☐ MRI-based neuroimaging

Plants

Seed stocks	Seeds were obtained from the Nottingham Arabidopsis Stock Centre (NASC), now the Eurasian Arabidopsis Stock Centre (https://arabidopsis.info/).
Novel plant genotypes	Novel plant genotypes were produced by genetic crossings. The characterization of the previously not fully characterized gai mutant of Arabidopsis is documented in Supplementary Figure 7.
Authentication	All genotypes were verified according to standard procedures using molecular markers which are listed in Supplementary Table 3. All genotypes used for experiments or genetic crossings were described previously (for references, see Supplementary Table 2) and showed no deviations from the published phenotypes under normal growth conditions.
